# Antimicrobial Drug Use and Resistance in Europe

**DOI:** 10.3201/eid1411.070467

**Published:** 2008-11

**Authors:** Nienke van de Sande-Bruinsma, Hajo Grundmann, Didier Verloo, Edine Tiemersma, Jos Monen, Herman Goossens, Matus Ferech, the European Antimicrobial Resistance Surveillance System and European Surveillance of Antimicrobial Consumption Project Groups

**Affiliations:** National Institute of Public Health and the Environment, Bilthoven, the Netherlands (N. van de Sande-Bruinsma, H. Grundmann, E. Tiemersma, J. Monen); University Medical Centre, Groningen, Groningen, the Netherlands (H. Grundmann); Veterinary and Agrochemical Research Centre, Ukkel, Belgium (D. Verloo); University of Antwerp, Antwerp, Belgium (H. Goossens, M. Ferech)

**Keywords:** Europe, surveillance, antimicrobial use, antimicrobial resistance, penicillin, erythromycin, fluoroquinolones, Streptococcus pneumoniae, Escherichia coli, research

## Abstract

Our study confronts the use of antimicrobial agents in ambulatory care with the resistance trends of 2 major pathogens, *Streptococcus pneumoniae* and *Escherichia coli,* in 21 European countries in 2000–2005 and explores whether the notion that antimicrobial drug use determines resistance can be supported by surveillance data at national aggregation levels. The data obtained from the European Surveillance of Antimicrobial Consumption and the European Antimicrobial Resistance Surveillance System suggest that variation of consumption coincides with the occurrence of resistance at the country level. Linear regression analysis showed that the association between antimicrobial drug use and resistance was specific and robust for 2 of 3 compound pathogen combinations, stable over time, but not sensitive enough to explain all of the observed variations. Ecologic studies based on routine surveillance data indicate a relation between use and resistance and support interventions designed to reduce antimicrobial drug consumption at a national level in Europe.

## Abstract 2

Routine surveillance data indicate a relation between use and resistance and support interventions designed to reduce antimicrobial consumption at a national level in Europe.

For the past 60 years, antimicrobial chemotherapy has been the mainstay of medical intervention against infectious diseases caused by bacterial pathogens. The continuous decline of therapeutic effectiveness as a result of extensive use of antimicrobial chemotherapy has been long predicted and seems inescapable (*1*). Many surveillance efforts have over the last decade (1997–2007) drawn attention to this phenomenon (*2*–*5*). At the same time, the once-abundant supply of new and improved antimicrobial compounds has worn thin, as drug development becomes increasingly challenging and pharmaceutical companies invest in more lucrative markets (*6*). It is therefore critical to realize that antimicrobial drug effectiveness, widely accepted as a common good, cannot be taken for granted and that such substances are increasingly attaining the status of nonrenewable resources.

Our study confronts the population-adjusted use of antimicrobial agents in ambulatory care with the resistance trends of 3 compound pathogen combinations in 21 European countries over a period of 6 years (2000–2005). This initial study was made possible by combining data from the 2 most comprehensive European surveillance systems on antimicrobial drug consumption and resistance, the European Surveillance of Antimicrobial Consumption (ESAC) (*7*) and the European Antimicrobial Resistance Surveillance System (EARSS) (*8*). We present an authoritative joint analysis of these 2 comprehensive databases. At this highly aggregated level, data are not sensitive enough to unravel the complex interaction between prescribing and resistance. The goal of this study is to give an overview of the situation in the European region and explore whether a relationship between antimicrobial drug use and resistance can be supported by empirical data pooled at national levels.

## Materials and Methods

### Consumption of Antimicrobial Agents

ESAC collects data on antimicrobial drug use in ambulatory care and hospital care in Europe. Currently, 24 countries report data on ambulatory care consumption to ESAC (*9*). Prescribed drugs are grouped by the active substance as the number of defined daily doses (DDD) per 1,000 inhabitants (DID) according to the World Health Organization definition of Anatomical Therapeutic Chemical Classification (ATC) defined daily dose (ATC-DDD version 2005 (*10*). A complete description of the data providers and details of the methods used by ESAC have been published (*7*,*11*,*12*). The performance and methodologic approach of the ESAC system, which aimed to collect comparable and reliable data on antimicrobial drug use, were studied by Vander Stichele et al. (*7*). The collected data were screened for bias caused by errors in assigning medicinal product packages to the ATC; errors in calculations of DDD per package; bias by over-the-counter sales and parallel trade; and bias in ambulatory care/hospital care mix. The study indicated that of the 31 participating countries, 21 delivered ambulatory care data suitable for cross-national comparison (*7*).

For the present study, the total country-specific antimicrobial drug use in ambulatory care and a breakdown into the following major antimicrobial classes were extracted from the ESAC database: penicillins (J01C); other β-lactam antimicrobial agents (cephalosporins, monobactams and carbapenems, J01D); macrolides, lincosamines, and streptogramins (MLS-class, J01F); and fluoroquinolones (J01MA).

### Resistance to Antimicrobial Agents

EARSS performs continuous surveillance of antimicrobial drug susceptibility for 7 major bacterial pathogens that cause invasive infections. Data are provided by >900 microbiologic laboratories that serve ≈1,400 hospitals from 32 countries with an overall hospital catchment population estimated to include >100 million inhabitants (*13*). All EARSS participating laboratories perform routine antimicrobial drug susceptibility tests according to standard protocols (*14*) and interpret their susceptibility results according to harmonized national and international guidelines as sensitive, intermediately resistant, and resistant (*15*). More details about the data acquisition and analysis have been published elsewhere (*13*,*16*,*17*). The antimicrobial susceptibility test (AST) results reported by the laboratories are collected by using standardized protocols as described in the EARSS manual (www.rivm.nl/earss). Data that do not meet the requirements of these species-specific protocols are not accepted. To assess the comparability of results between laboratories participating in EARSS, an external quality assessment exercise is organized every year. A set of 6 strains is provided to each laboratory in collaboration with the UK National External Quality Assurance Scheme. These exercises illustrate that routinely reported results, as collected by EARSS, have sufficient accuracy to provide good estimates of overall resistance prevalences and trends (*18*).

For the present study, AST results of primary blood culture isolates of *Escherichia coli* and *Streptococcus pneumoniae* were extracted from the EARSS database to determine the proportions of penicillin- and erythromycin-nonsusceptible *S. pneumoniae* (PNSP and ENSP, respectively) and proportions of fluoroquinolone-resistant *Escherichia coli* (FQRE) bacteria. Nonsusceptible isolates included both intermediate resistant and resistant isolates. A country-specific resistance score was calculated as the sum of the quartile ranks of resistance against all 3 compound pathogen combinations (PNSP, ENSP, and FQRE). For trend analysis of resistance proportions per country over time, the Cochrane-Armitage trend test was used.

### Ecologic Analysis

The strength of association between antimicrobial drug use and resistance was determined by univariate and multiple linear regression analysis. The proportion of resistance (R) in a country was transformed to the natural logarithm of the odds of resistance (ln[R/1–R]), to get a range from –∞ to +∞. The log odds of resistance (as the dependent variable) can then be expressed as a simple linear function of the independent variable (consumption) (*19*,*20*). To give equal weight to small countries with flawless data collection and not give the unequal weight to larger countries with sometimes less-optimal data, the linear regression analysis was not weighed.

To determine the delay between antimicrobial use and resistance, proportions of PNSP, ENSP, and FQRE for 2002–2005 were correlated with the consumption of different antimicrobial drug classes in the same year and the 2 years before. This resulted in 11 different exposure-outcome intervals for each compound–pathogen combination. For further multivariate analysis, the interval with the median correlation coefficient was regarded as representative for the association found in the overall study period.

Only the countries that reported volumes of antimicrobial drug prescriptions in ambulatory care from 2000 through 2004 and susceptibility data for the selected compound–pathogen combinations from 2002 through 2005 were included for linear regression analysis. Countries that provided yearly susceptibility data for <20 isolates were excluded. Data analysis was conducted by using SAS version 9.1 software (SAS Institute Inc., Cary, NC, USA).

## Results

### Consumption of Antimicrobial Agents

**Figure 1:**
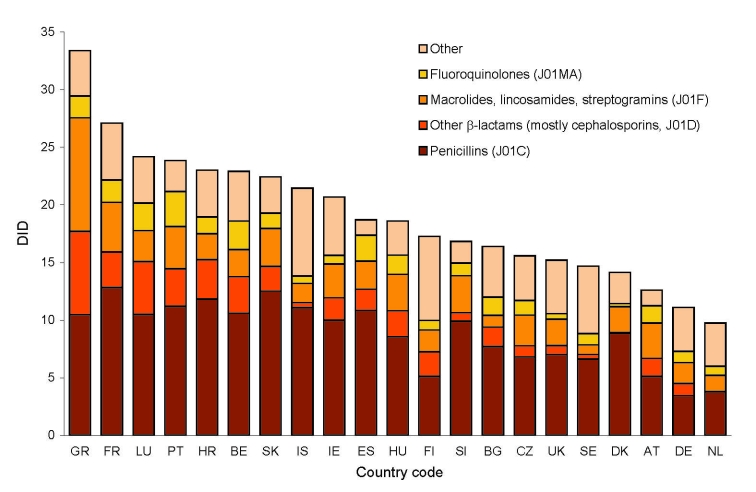
Total antimicrobial drug consumption in ambulatory care in defined daily dose per 1,000 inhabitants per day (DID) by antimicrobial class in 21 European countries in 2004. See Table 1 footnote for country designations.

We included in the study 21 European countries, which provided data on the use of antimicrobial agents in ambulatory care to the ESAC database for the period 2000–2004 (including the 15 long-standing European Union (EU) member states). These also included 3 of the 10 nations that joined the EU in May 2004, the Czech Republic, Slovakia, and Slovenia; 2 applicant countries, Bulgaria and Croatia; and 1 European Free Trade Association country, Iceland. Total outpatient antimicrobial drug use differed significantly between countries. Use tends to be low in northern, moderate in central, and high in southern Europe and varied by a factor of 3.4 between Greece (33.4 DID) and the Netherlands (9.7 DID) in 2004 (Figure 1, Table 1).

**Table 1:** Difference in outpatient antimicrobial drug use DID in 21 European countries, 2004, and changes in use, 2000–2004*†

Substance class (ATC category)	Antimicrobial use, DIDs, 2004				Changes in antimicrobial drug use, 2000–2004	
	Maximum (country)	Minimum (country)	fd		>15% increase	>15% decrease
Total use (J01)	33.4(GR)	9.7 (NL)	3.4		HU, DK, GR, IE	BG, CZ, DE, FR
Penicillins (J01C)	12.8 (FR)	3.4 (DE)	3.8		HU, DK	CZ, FR, DE, SK
Cephalosporins, monobactams, carbapenems (J01D)	7.2 (GR)	0.05 (NL)	>100		SI	BE, BG, CZ, FR, IS NL, ES, SE
Macrolides, lincosamines, streptogramins (J01F)	9.9 (GR)	0.8 (BG)	12.4		BG, HR, GR, IE, NL	BE, FR, DE, LU, ES
Fluoroquinolones (J01MA)	3.04 (PT)	0.28 (DK)	10.9		AT, BG, CZ, DK, FI, DE, HU, IE, LU, UK	SI

*DID, defined daily dose/1,000 inhabitants; ATC, Anatomic Therapeutic Chemical classification; fd, factor difference. †Country designations: AT, Austria; BE, Belgium; BG, Bulgaria; CZ, Czech Republic; DE, Germany; DK, Denmark; ES, Spain; FI, Finland; FR, France; GR, Greece; HR, Croatia; HU, Hungary; IE, Ireland; LU, Luxembourg; NL, the Netherlands; PT, Portugal; SE, Sweden; SI, Slovenia; SK, Slovakia; UK, United Kingdom.

During the observation period (2000–2004), antimicrobial drug use decreased (>15%) in Bulgaria, Czech Republic, France, and Germany and increased (>15%) in Croatia, Denmark, Greece, and Ireland. Penicillins (including broad-spectrum penicillins, ATC category J01C) represented the most widely used antimicrobial class in Europe. This class showed consumption patterns similar to the total outpatient antimicrobial drug use, as did the second most widely used category, which consists mainly of macrolides but also includes lincosamines and streptogramins (MLS class, ATC category J01F). The third most widely used ATC category (J01D, other β-lactams) consists of cephalosporins, monobactams, and carbapenems. Cephalosporins make up the bulk of the antimicrobial agents included in this group. Antimicrobial agents belonging to this category are more commonly used in hospitals; however, in some countries they are also extensively prescribed in ambulatory care. For this reason, use rates in Europe varied >100-fold between countries. The use of this ATC category decreased by >15% in 8 countries but increased in Slovenia. Fluoroquinolones hold the fourth position in the European market but showed the most dynamic increase, with growth rates of >15% in almost half of all countries (10/21). In terms of overall control of antimicrobial drug consumption, France most consistently reduced its use of 3 of the 4 most frequently prescribed antimicrobial drug classes (Figure 1, Table 1).

### Resistance to Antimicrobial Agents

**Figure 2:**
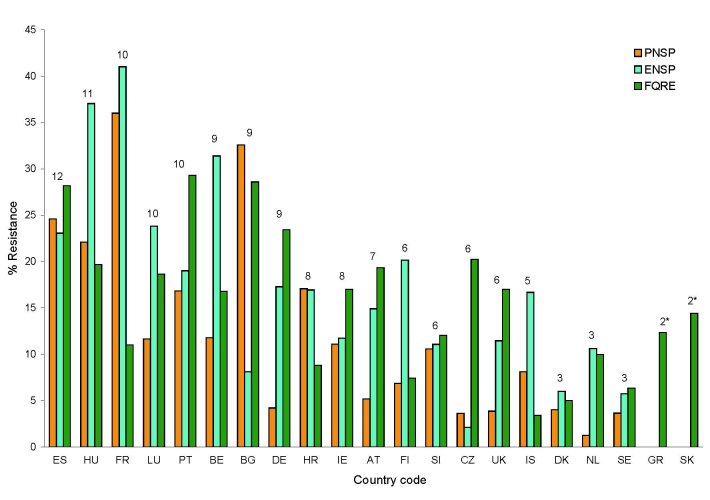
Proportion of penicillin-nonsusceptible *Streptococcus pneumoniae* (PNSP), erythromycin-nonsusceptible *S. pneumoniae* (ENSP), and fluoroquinolone-resistant *Escherichia coli* (FQRE) in 2005, ranked in descending order by country-specific resistance score indicated above bars. *For Greece and Slovakia, data on *S. pneumoniae* resistance were not available. Country (total no. of *S. pneumoniae* isolates reported/ total no. of *E. coli* isolates reported): ES (740/2993); HU (86/468); FR (632/6028); LU (43/188); PT (202/1086); BE (1539/1461); BG (43/196); DE (119/957); HR (129/637); IE (397/1411); AT (290/2049); FI (525/1743); SI (208/657); CZ (194/2233); UK (1373/2359); IS (23/46); DK (1081/1283); NL (802/2140); SE (1017/3035); GR (0/1136); SK (0/132). See Table 1 footnote for country designations.

Large differences in the proportions of resistance were reported for the same countries. The highest antimicrobial drug resistance was found in Spain, Hungary, and France and the lowest in Sweden and the Netherlands in 2005 (Figure 2). Resistance proportions in 2005 differed by a factor of 27.7 for PNSP between France (36%) and the Netherlands (1.3%), by 20.5 for ENSP between France (41%) and the Czech Republic (2%), and by 9.7 for fluoroquinolone resistance in *E. coli* between Portugal (29%) and Iceland (3%). From 2001 through 2005, resistance levels remained relatively stable for PNSP but increased for the other 2 compound pathogen combinations (Table 2). Spain and the United Kingdom were the only countries that reported any significant decrease in antimicrobial drug resistance rates. In Spain, penicillin nonsusceptibility fell from 37% to 25% and in the United Kingdom, from 5% to 3.8%. For ENSP a significant increase was observed in Hungary (from 19% to 37%), Finland (from 12% to 20%), and the Netherlands (5% to 11%). The most consistent trend was observed for fluoroquinolone resistance in *E. coli*, which increased in most European countries (Table 2).

**Table 2:** Differences in the proportion of antimicrobial drug resistance in 21 European countries, 2005, and significant trends, 2001–2005

Compound-pathogen†	Antimicrobial drug resistance					
	Europe, %, 2005				Trends, 2001–2005*	
	Maximum (country)	Minimum (country)	fd‡		Increase (p<0.05)	Decrease (p<0.05)
PNSP	36 (FR)	1.3 (NL)	27.7		BG	ES, UK
ENSP	41 (FR)	2 (CZ)	20.5		FI, HU, NL	
FQRE	29 (PT)	3 (IS)	9.7		AT, BE, BG, CZ, DE, ES, FI, HR, HU, LU, NL, PT, SE	

*No trend analysis was performed for Denmark and France, and for Ireland and the United Kingdom for proportion of *Escherichia coli* resistant to fluoroquinolones (FQRE), because data were not available for all years of the study period (2001–2005). See Table 1 footnote for country designations. †PNSP, proportion of *Streptococcus pneumoniae* not susceptible to penicillin; ENSP, erythromycin-nonsusceptible *S. pneumoniae*. ‡fd, factor difference.

### Combining Antimicrobial Drug Use with Susceptibility Data

Greece (33.0 DID), France (27.1 DID), Luxembourg (24.2 DID), Portugal (23.8 DID), Croatia (23.0 DID), and Belgium (22.9 DID) were the countries that reported the highest use of antimicrobial agents in ambulatory care. Four of these high-consumer countries—France, Luxemburg, Belgium, and Portugal—were also among the 6 countries with the highest resistance proportions. Croatia occupied an intermediate resistance rank, owing to more modest levels in fluoroquinolone resistance. For Greece, susceptibility data for *S. pneumoniae* were not available, which precluded a meaningful ranking. Although Spain (18.7 DID) and Hungary (18.6 DID) were not among the countries with the highest use of antimicrobial agents, both countries did have the highest antimicrobial drug resistance proportions in 2005. The United Kingdom (15.2 DID), Sweden (15 DID), Denmark (14.1 DID), Austria (12.5 DID), Germany (11 DID), and the Netherlands (10 DID) reported the lowest antimicrobial drug use in outpatient settings. Of these, Sweden, the Netherlands, Denmark, and the United Kingdom also were among the 6 countries with the lowest resistance proportions. Germany and Austria reported medium to high rates especially for ENSP (17% and 15%, respectively) and FQRE (23% and 19%, respectively) (Figures 1, 2). Because inspection of the data suggested a relation between antimicrobial drug consumption and resistance, this assumption was formally tested by using simple linear regression.

Because little is known about the delay that can be expected between the change in antimicrobial drug exposure and its effect on antimicrobial resistance at a population level, different intervals were chosen to explore the potential association between use and resistance. Intervals were explored for same-year data, a 1-year delay, and a 2-year delay between exposure and outcome. Thus, the consumption data available for 2000 through 2004 and resistance data for 2002–2005 provided the means to explore the correlation coefficients of 11 exposure-outcome intervals. Only the 17 countries that provided data for all years were included in the linear regression analysis. Table 3 shows the range and median correlation coefficient for all exposure-outcome intervals. Since no statistically significant time dependence was observed, the median correlation coefficient was regarded as representative for the association found for the entire study period (Table 3).

**Table 3:** Range and median correlation between the occurrence (logodds) of PNSP, ENSP, and FQRE in 2002–2005 and antimicrobial drug consumption, Europe, 2000–2004*

E consumption	O-resistance phenotype	No. E–O intervals with significant association†	Correlation coefficients (*r*)							
			Median			Minimum			Maximum	
			*r* (CI)	E–O year		*r* (CI)	E–O year		*r* (CI)	E–O year
Total use (J01)	PNSP	11	0.68 (0.30–0.87)	2003–2003		0.61 (0.17–0.84)	2001–2003		0.73 (0.39–0.90)	2002–2002
	ENSP	9	0.55 (0.07–0.82)	2001–2003		0.37 (–0.11 to 0.75)	2004–2005		0.71 (0.33–0.89)	2003–2003
Penicillins (J01C)	PNSP	11	0.78 (0.48–0.92)	2003–2004		0.69 (0.28–0.87)	2003–2005		0.82 (0.55–0.93)	2004–2004
	ENSP	3	0.37 (–0.15 to 0.74)	2003–2005		0.26 (–0.29 to 0.66)	2001–2002		0.60 (0.15–0.84)	2003–2003
Cephalosporins, monobactams, carbapenems (J01D)	PNSP	8	0.57 (0.13–0.83)	2002–2003		0.41 (–0.07 to 0.74)	2002–2004		0.64 (0.23–0.86)	2000–2002
	ENSP	11	0.69 (0.30–0.88)	2001–2002		0.50 (0.00–0.79)	2003–2005		0.79 (0.48–0.92)	2004–2004
Macrolides, lincosamides, streptogramins (MLS class J01F)	PNSP	4	0.42 (–0.08 to 0.75)	2004–2004		0.26 (–0.22 to 0.67)	2004–2005		0.53 (0.07–0.81)	2002–2002
	ENSP	9	0.56 (0.08–0.82)	2001–2002		0.35 (–0.19 to 0.71)	2004–2004		0.67 (0.27–0.88)	2003–2004
Fluoroquinolones (JO1MA)	PNSP	9	0.51 (0.04–0.80)	2004–2004		0.36 (–0.10 to 0.74)	2003–2005		0.57 (0.12–0.82)	2002–2002
	ENSP	10	0.62 (0.18–0.85)	2001–2002		0.48 (–0.04 to 0.78)	2004–2005		0.69 (0.29–0.89)	2004–2004
	FQRE‡	9	0.60 (0.17–0.84)	2004–2004		0.44 (–0.05 to 0.76)	2003–2005		0.70 (0.33–0.88)	2001–2002

*PNSP, penicillin-nonsusceptible *Streptococcus pneumoniae;* ENSP, erythromycin-nonsusceptible *S. pneumoniae*; FQRE, fluoroquinolone-resistant *Escherichia coli*; E, exposure; O, outcome; CI, 95% confidence interval; MLS, macrolides, lincosamines, and streptogramins. †Exposure outcome intervals include all 11 possible time windows, considering the data for consumption (exposure) and resistance (outcome) for the same year as well as for intervals of 1 to 2 y between exposure and outcome. p<0.05 was significant. ‡Significant correlations of fluoroquinolone consumption were found only with FQRE. Other correlations were therefore not shown.

**Figure 3:**
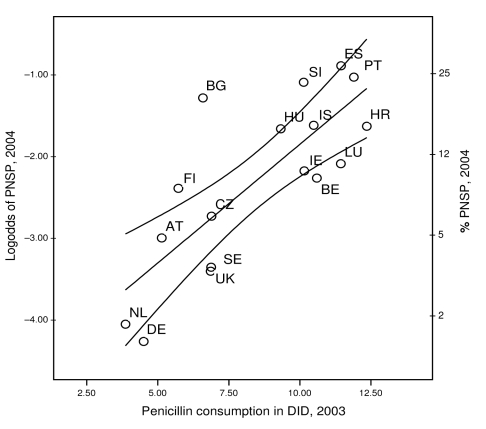
Occurrence of penicillin-nonsusceptible *Streptococcus pneumoniae* (PNSP) plotted against outpatient use of penicillins in 17 European countries including 95% confidence intervals. DID, defined daily doses per 1,000 inhabitants. See Table 1 footnote for country designations.

The occurrence of PNSP in European countries correlated with the country-specific use of penicillins, which explained 61% of the observed variance (p<0.01) (Figure 3). The second best correlation was provided by the total antimicrobial drug use in ambulatory care, which explained 46% of the observed variance (p<0.01). Both associations were robust and remained significant, regardless of the interval between the ascertainment of antimicrobial drug use and the recording of antimicrobial resistance. A notably less consistent association was found when we correlated the use of MLS-class antimicrobial agents or fluoroquinolones with the occurrence of PNSP (Table 3). ENSP occurrence in Europe correlated most compellingly with the country-specific use rate of ATC category J01D (other β-lactams), which explained 48% of the observed variance (p<0.01) (Table 3). However, this effect appeared to be confounded by the use of MLS-class antimicrobial agents and fluoroquinolones. By fitting use data for these antimicrobial agents into the model, the effect estimates for the former decreased by 40% (Table 4), indicating that part of the effect attributed to the use of other β-lactam antimicrobial agents appeared to be exerted by MLS-class antimicrobial agents and fluoroquinolones.

**Table 4:** Results of multiple linear regression for the occurrence of PNSP and ENSP*

Models	Outcome variable: logodds PNSP			
	Exposure	Parameter estimate	p value	*R* ^2^
Model 1				
Intercept		–4.75		
Gradient	Penicillins	0.29	0.0002	0.61
Model 2				
Intercept		–4.8		
Gradient	Penicillins	0.33	0.002	
	Other β-lactams	–0.05	0.808	
	Fluoroquinolones	–0.11	0.73	0.62
	Outcome variable: logodds ENSP			
Model 1				
Intercept		–2.82		
Gradient	Other β-lactams	0.41	0.003	0.48
Model 2				
Intercept		–3.26		
Gradient	Other β-lactams	0.25	0.14	
	MLS class	0.15	0.39	
	Fluoroquinolones	0.30	0.35	0.56

*PNSP, penicillin-nonsusceptible *Streptococcus pneumoniae*; ENSP, erythromycin-nonsusceptible *S. pneumoniae*; MLS, macrolides, lincosamines, and streptogramins.

**Figure 4:**
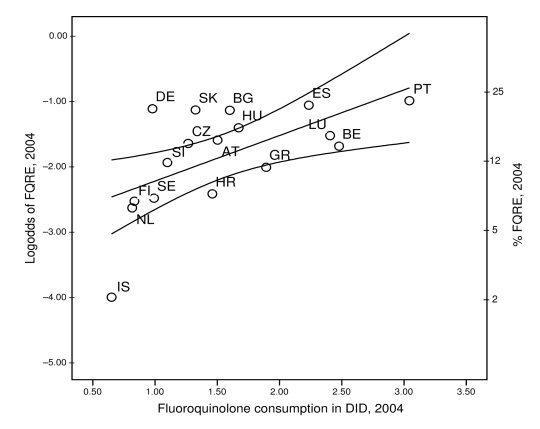
Occurrence of fluoroquinolone-resistant *Escherichia coli* (FQRE) plotted against outpatient use of fluoroquinolone antimicrobial agents in 17 European countries including 95% confidence intervals. DID, defined daily doses per 1,000 inhabitants. See Table 1 footnote for country designations.

Proportions of FQRE in European countries were best explained by the country-specific use data for fluoroquinolones. Fluoroquinolone consumption as reported to the ESAC network explained 36% of the variance observed in EARSS data (p<0.01; Figure 4). This effect appeared to be specific and was not associated or confounded by consumption of the other antimicrobial classes.

## Discussion

We compared the trends in antimicrobial drug consumption patterns and the antimicrobial drug resistance proportions for 2 major pathogens, *S. pneumoniae* and *E. coli,* in Europe from 2000 through 2005. Antimicrobial drug use in outpatient settings was ascertained by the most comprehensive network for European surveillance of antimicrobial consumption (ESAC), and antimicrobial resistance data were obtained from the European surveillance system EARSS). The data suggested that in Europe the variation of consumption coincides with the occurrence of resistance at country level. Using simple linear regression analysis, we formally explored whether a relation between country-specific antimicrobial drug use and antimicrobial resistance can be inferred at national aggregation levels and found that the association between antimicrobial drug use and resistance was specific and robust for 2 of the 3 compound pathogen combinations under study, stable over time, but not sensitive enough to explain all of the observed variation.

There was a high degree of consistency between penicillin use and penicillin nonsusceptibility in pneumococci as well as for fluoroquinolone use and an increase in fluoroquinolone resistance in *E. coli.* Simple linear regression showed that these effects were highly specific and robust, as inclusion of the use of other antimicrobial substances did not improve correlation or was not confounding the overall effect estimates (Tables 3, 4; Figures 3, 4). The mechanisms for acquiring resistance against both substances have some features in common. These include successive alterations of chromosomally located genes by either homologous recombination or point mutations, resulting in a stepwise modification of the molecular targets, which first leads to reduced susceptibility and eventually to complete resistance (*21*,*22*). In contrast to many other resistance mechanisms, no mobile genetic elements are involved, and a physical linkage to other resistance determinants is unlikely. It is therefore expected that before phenotypes with stable combined resistance evolve, antimicrobial drug selection will specifically favor homologous resistance.

A nonhomologous effect was observed in the case of ENSP, since the variance in ENSP occurrence was best explained by the country-specific use rates of the ATC category of other β-lactams, consisting mainly of cephalosporins. This observation could be either causal, coincidental, or both. In fact, the results of multiple regression models indicate a degree of confounding, as part of the effect attributed to other β-lactams could be explained by MLS-class antimicrobial agents and fluoroquinolones (Table 4). This confounding effect implies that the effect of other β-lactams is mixed with the effect of MLS-class antimicrobial agents and fluoroquinolones used. Data recorded by ESAC suggest that most countries with high use of other β-lactams also have a high consumption of MLS-class antimicrobial agents (*r* = 0.78, p<0.01) as well as fluoroquinolones (*r* = 0.65, p<0.01). Moreover, countries with the highest levels of other β-lactam use—such as Luxembourg, Croatia, Portugal, Belgium, and France—and high levels of ENSP (23%, 19%, 20%, 31%, and 41%) also reported high levels of combined nonsusceptibility to both erythromycin and penicillin (12%, 9%, 10%, 9%, and 32%). Any increase in selection pressure exerted by β-lactams would also co-select for ENSP under these conditions of combined nonsusceptibility, which could also explain the absence of a direct relationship between use of MLS-class antimicrobial agents and ENSP.

For all compound pathogen combinations that showed significant correlations, the association between the volume of antimicrobial agents used and proportions of resistance was for the most part stable, i.e., independent of the time lag between recording of consumption and the recording of resistance (Table 3). This is not surprising because in the absence of nationwide interventions that would abruptly change the use pattern for an entire country, no major trend changes would be expected, or as other authors have already stated, it is likely that a country with more use or resistance than others in one year, will also have more use or resistance in the next (*19*). Likewise, the steady decline in the consumption in some of the antimicrobial drug classes such as penicillins, as happened in the Czech Republic, France, Germany, and Slovakia, was not reflected by a concomitant decline of penicillin resistance in the pathogens under selective pressure. Mathematical models as well as empirical data suggest that after a reduction in prescribing, resistance will take longer to decline than it took to rise (*23*). In the same way, no decline in resistance against co-trimoxazole was observed in the United Kingdom even 10 years after it abandoned its prescribing, which in this instance was attributed to the co-selection of genetically linked resistance determinants by alternative antimicrobial pressure (*24*). EARSS data show a significant reduction of penicillin resistance in Spain (*25*) and the United Kingdom over the past 5 years (2001–2005), however, no corresponding decline in penicillin use has become apparent that could explain this favorable development (Tables 1, 2). Alternatively, data aggregated at country level by established surveillance networks may not be sensitive enough to identify subtle changes in the complex interaction between antimicrobial drug prescribing and resistance.

EARSS data consist of antimicrobial drug resistance proportions of bacteria that cause invasive bloodstream infections but do not include information from other potentially relevant patient materials. This omission limits the wealth of data but improves the comparability between participating laboratories because it reduces bias introduced by differential case ascertainment. *S. pneumoniae* is the main cause of community-acquired bacteremic pneumonia (*26*), and invasive *E. coli* infections are mainly caused by the translocation of intestinal colonizing strains (*27*). Thus, we believe that resistance among *S. pneumoniae* and *E. coli* blood culture isolates would sufficiently reflect the ecological pressure exerted by the antimicrobial drug use in outpatient settings.

There is little doubt that antimicrobial drug consumption is important in the dissemination of antimicrobial drug resistance. However, additional or alternative factors need to be taken into account (*28*).

We could not control for country-specific differences in hygiene, diagnostic habits, community infection control, and vaccination policies that could provide alternative explanations for some of the observed differences. Moreover, inconsistencies in the sampling population covered by the 2 surveillance systems may introduce inaccuracies that hamper the internal validity of this type of analysis (*29*). In general, data at this high aggregation level are probably not sensitive enough to reflect subtle changes in the complex interaction between antimicrobial drug prescribing and resistance. In this respect, increasing the geographic resolution of data collection by addressing antimicrobial drug use and resistance at the level of health districts would improve the analysis and degree of causal inference that these studies could provide. A higher geographic resolution could also foster interventions by making local extremes of use apparent. However, despite these drawbacks, the data suggest that a specific, robust, and stable association exists between antimicrobial drug use and the occurrence of resistance at country level in the European Union. Our results therefore support interventions that encourage healthcare professionals and healthcare authorities to take firm steps toward promoting prudent use and careful restriction of antimicrobial drug prescription and to monitor the effect of these interventions toward the restoration of the antiinfective activity essential to the success of modern medicine.
